# Viral genome sequence datasets display pervasive evidence of strand-specific substitution biases that are best described using non-reversible nucleotide substitution models

**DOI:** 10.21203/rs.3.rs-2407778/v1

**Published:** 2022-12-29

**Authors:** Rita Sianga-Mete, Penelope Hartnady, Wimbai Caroline Mandikumba, Kayleigh Rutherford, Christopher Brian Currin, Florence Phelanyane, Sabina Stefan, Sergei L Kosakovsky Pond, Darren Patrick Martin

**Affiliations:** University of Cape Town; University of Cape Town; University of Cape Town; University of Cape Town; University of Cape Town; University of Cape Town; Brown University; Temple University; University of Cape Town

**Keywords:** Reversibility, Non-reversibility, Mutations, Models of evolution

## Abstract

**Background:**

The vast majority of phylogenetic trees are inferred from molecular sequence data (nucleotides or amino acids) using time-reversible evolutionary models which assume that, for any pair of nucleotide or amino acid characters, the relative rate of X to Y substitution is the same as the relative rate of Y to X substitution. However, this reversibility assumption is unlikely to accurately reflect the actual underlying biochemical and/or evolutionary processes that lead to the fixation of substitutions. Here, we use empirical viral genome sequence data to reveal that evolutionary non-reversibility is pervasive among most groups of viruses. Specifically, we consider two non-reversible nucleotide substitution models: (1) a 6-rate non-reversible model (NREV6) in which Watson-Crick complementary substitutions occur at identical relative rates and which might therefor be most applicable to analyzing the evolution of genomes where both complementary strands are subject to the same mutational processes (such as might be expected for double-stranded (ds) RNA or dsDNA genomes); and (2) a 12-rate non-reversible model (NREV12) in which all relative substitution types are free to occur at different rates and which might therefore be applicable to analyzing the evolution of genomes where the complementary genome strands are subject to different mutational processes (such as might be expected for viruses with single-stranded (ss) RNA or ssDNA genomes).

**Results:**

Using likelihood ratio and Akaike Information Criterion-based model tests, we show that, surprisingly, NREV12 provided a significantly better fit to 21/31 dsRNA and 20/30 dsDNA datasets than did the general time reversible (GTR) and NREV6 models with NREV6 providing a better fit than NREV12 and GTR in only 5/30 dsDNA and 2/31 dsRNA datasets. As expected, NREV12 provided a significantly better fit to 24/33 ssDNA and 40/47 ssRNA datasets. Next, we used simulations to show that increasing degrees of strand-specific substitution bias decrease the accuracy of phylogenetic inference irrespective of whether GTR or NREV12 is used to describe mutational processes. However, in cases where strand-specific substitution biases are extreme (such as in SARS-CoV-2 and Torque teno sus virus datasets) NREV12 tends to yield more accurate phylogenetic trees than those obtained using GTR.

**Conclusion:**

We show that NREV12 should, be seriously considered during the model selection phase of phylogenetic analyses involving viral genomic sequences.

## Background

Modelling the nucleotide substitution processes that underly the diversification of virus genome sequences is at the heart of many viral evolutionary analyses. The most widely used nucleotide substitution models belong to the general time reversible (GTR) family(Tavaré, 1986) that assume that the Markov process of evolution will occur in the same way both forward and backward in time such that, when the arrow of time is inverted, the forward process cannot be distinguished from the backward process (Hoff, Orf, Riehm, Darriba, & Stamatakis, 2016), (Lio & Goldman, 1998),(Tavaré, 1986).

The essence of the GTR model is captured in the definition of its instantaneous rate matrix in Eq. (1); a matrix that models the rates at which the four different nucleotides {A,C,G,T} are exchanged, ensuring that the detailed reversibility balance condition: $Q_{ji}\pi_{i}=Q_{ij}\pi_{j}$ (where *Q*_*ji*_ is the instantaneous rate of change from *j* to *i* and $\pi_{i}$ is the equilibrium probability of state *i*) is met(Squartini & Arndt, 2008) (Posada, 2003). The instantaneous rate matrix of the GTR model consists of six parameters (a, b, c, d, e, and f) which indicate the relative rates from base *i* to *j* in the state space {A, C, G, T} and the $\pi_{i}$‘s are the equilibrium frequencies of each base

(1) $$Q=\left\{q_{ij}\right\}=\left(\begin{array}{cccc} - & a\pi_{A} & b\pi_{A} & d\pi_{A}\\ a\pi_{C} & - & c\pi_{C} & e\pi_{C}\\ b\pi_{G} & c\pi_{G} & - & f\pi_{G}\\ d\pi_{T} & e\pi_{T} & f\pi_{T} & - \end{array}\right)$$

The rate matrix in Eq. (1) is symmetrical in that, for example, the relative rate at which A changes to G is the same as the relative rate at which G changes to A.

Time reversible nucleotide substitution models such as GTR form the basis of almost all nucleotide sequence-focused evolutionary analyses (including those involving eukaryotes, prokaryotes, and viruses) (Lefort, Longueville, & Gascuel, 2017), (Posada & Crandall, 2001), (Minin, Abdo, Joyce, & Sullivan, 2003).

The reliability of a phylogenetic tree constructed using a particular nucleotide sequence dataset should be maximized when the evolutionary models used to construct the tree accurately reflect the evolutionary processes that yielded the nucleotide sequence dataset (Buckley & Cunningham, 2002), (Ripplinger & Sullivan, 2008), (Hoff, Orf, Riehm, Darriba, & Stamatakis, 2016). The suitability of different models for describing the evolution of DNA or RNA sequences is, therefore, expected to depend to some degree on the biological and environmental contexts of the sequences being analyzed.

Mutations in viral genomes arise due to diverse biotic (such as replication enzyme infidelities, RNA/DNA editing enzymes) and abiotic (such as ionizing radiation, inorganic oxidizers and chemical mutagens) factors (Sanjuán & Domingo-Calap, 2016). Mutagenic chemical reactions or types of radiation that, for example, cause G to A or C to U mutations in DNA or RNA, are not the same as those that cause A to G or U to C mutations (Cheng, Cahill, Kasai, Nishimura, & Loeb, 1992), (Nguyen, et al., 1992), (Chelico, Pham, Calabrese, & Goodman, 2006), (Sharma, Patnaik, Taggart, & Baysal, 20016). It should not be expected, therefore, that the relative rates of G to A substitution will equal as the relative rates of A to G substitution. Instead, in evolving double-stranded (ds) DNA and dsRNA molecules where both strands of the genome are in existence for similar amounts of time, both G to A and C to T substitutions should occur at relatively similar rates. Therefore, for nucleotide sequence datasets derived from any organisms with dsDNA or dsRNA genomes, a non-reversible nucleotide substitution model with a different relative substitution rate category for each of the six possible pairs of complementary nucleotide substitutions (e.g. NREV6 in Eq. (2)), might plausibly provide a better description of mutational processes than GTR (Baele, Van de Peer, & Vansteelandt, 2010), (Wickner, 1993).


(2) $$Q=\left\{q_{ij}\right\}=\left(\begin{array}{cccc} - & a\pi_{A} & b\pi_{A} & c\pi_{A}\\ f\pi_{C} & - & d\pi_{C} & e\pi_{C}\\ e\pi_{G} & d\pi_{G} & - & f\pi_{G}\\ c\pi_{T} & b\pi_{T} & a\pi_{T} & - \end{array}\right)$$

In the case of single-stranded (ss) RNA viruses, ssDNA viruses, retroviruses, and dsRNA/dsDNA viruses where the two complementary genome strands do not exist for equal amounts of time (Yu, et al., 2004), a model where all twelve different substitutions are free to occur at different rates might be best. Specifically, with ssRNA viruses, ssDNA viruses, and retroviruses, only one of the genome strands (called the virion strand) is packaged into viral particles for transmission and, in many dsRNA viruses, the genome strand that is translated into proteins (called the + strand) exists for longer during the life cycle than does the complementary (or −) strand (Bruslind, 2020), (Onwubiko, et al., 2020). In all these viruses, some degree of strand-specific substitution bias is expected to occur (Van Der Walt, Martin, Varsani, Polston, & Rybicki, 2008), (Polak & Arndt, 2008) such that NREV6 might be anticipated to provide a poorer description of mutational processes than a model such as NREV12 (Eq. (3)) where each of the twelve different types of substitution has a different relative rate (Baele, Van de Peer, & Vansteelandt, 2010).


(3) $$Q=\left\{q_{ij}\right\}=\left(\begin{array}{cccc} - & a\pi_{A} & b\pi_{A} & c\pi_{A}\\ g\pi_{C} & - & d\pi_{C} & e\pi_{C}\\ h\pi_{G} & i\pi_{G} & - & f\pi_{G}\\ j\pi_{T} & k\pi_{T} & l\pi_{T} & - \end{array}\right)$$

Because non-reversible models consider the directionality of evolution, they could, in some cases, be used to identify root nodes of phylogenetic trees (Yap & Speed, 2005), (Boussau & Gouy, 2006). It is, however, unclear whether non-reversible models might, in certain situations at least, perform better than reversible models in the context of phylogenetic inference. Although it is possible to use non-reversible nucleotide substitution models such as NREV6 and NREV12 during maximum likelihood-based phylogenetic inference with computer programs such as IQ-TREE (Nguyen, Schmidt, Von Haeseler, & Minh, 2015), these models are still not routinely used for phylogenetic inference. This is in part because non-reversible models render several commonly used algorithmic techniques for efficient likelihood computation inapplicable. It is also in part because it remains undetermined whether, under conditions where strand-specific substitution biases are evident, non-reversible models consistently yield substantially more accurate phylogenetic trees than reversible models.

Here we present evidence that strand-specific nucleotide substitution biases are common within virus genomic sequence datasets such that NREV12 generally provides a significantly better fit than both GTR and NREV6 for such datasets. We then use simulations to demonstrate that whereas strand-specific nucleotide substitution biases reduce the accuracy of phylogenetic inference irrespective of whether GTR or NREV12 are used, when these biases become extreme use of NREV12 can yield significantly more accurate phylogenetic trees than GTR.

## Materials And Methods

### Virus sequence datasets and phylogenetic trees.

We obtained nucleotide sequences from the National Centre for Biotechnology Information Taxonomy database (http://www.ncbi.nlm.nih.gov/taxonomy) and the Los Alamos National Laboratory HIV sequence database (https://www.hiv.lanl.gov/content/index). These included gene and whole-genome sequences for viruses with ssRNA, ssDNA, dsRNA, and dsDNA genomes (datasets are summarized in supplementary Table S1). An outgroup sequence from a closely related virus species was added to each dataset to help root phylogenetic trees accurately. The sequences in each of the datasets were aligned using MUSCLE (Edgar, 2004) implemented in Aliview (Larsson, 2014), and maximum likelihood phylogenetic trees were constructed from each alignment using RAxML v8.2 (Stamatakis, 2016).

### Model Testing.

We evaluated the fit of NREV12, NREV6, and GTR to the 141 individual sequence datasets using a previously published model test (Harkins, et al., 2009) formulated in the HyPhy scripting language (Pond, Frost, & Muse, 2005). This script (which can be obtained from https:/github.com/veg/hyphy-analyses/tree/master/NucleotideNonREV) took as input a rooted maximum likelihood phylogenetic tree (minus the rooting sequence) and its corresponding nucleotide sequence alignment. The first step of the model testing process involved the harvesting of nucleotide sequences $\left(\frac{\pi_{i}}{\sum_{\text{i}=1}^{4}\pi_{i}}\right)$ from the sequence alignment into a vector of frequencies $\pi =\left\{\pi_{\text{A}},\pi_{\text{C}},\pi_{\text{G}},\pi_{\text{T}}\right\}$ called the frequency distribution matrix containing three free parameters with that of nucleotide T conditioned at absolute $\left(1-\pi_{\text{A}}-\pi_{\text{C}}-\pi_{\text{G}}\right)$.

Once the frequencies were harvested, the first stochastic rate matrix consisting only of the relative rates picked from a gamma distribution was defined to satisfy the reversibility conditions of relative rates being equal in reverse i.e., rAG = rGA, rAC = rCA, rAT = rTA, rCG = rGC, rTG = rGT, rCT = rTC. Thereafter the instantaneous rate matrix Q was calculated by multiplying the relative substitution rates by the appropriate nucleotide frequencies which were used to form the GTR model probability transition matrix, P by P (t) = e^Qt^. The role of the GTR model during model testing was to model mutations along the branches of tree, T. Given parameters of the GTR model describing (1) equilibrium nucleotide frequencies, and (2) the nucleotide substitution process, the likelihood, L, of the observed data, D, was calculated with the values of all the independent parameters, Ѳ, being investigated to find the combination that maximized the value of L (maximizing L ( ∣ D, T). The value of the log likelihood (lnL) under the GTR model was, at this point, stored for future comparisons with those of the NREV6 and NREV12 models.

The model was then changed to one satisfying the complementary relative reversibility conditions of the NREV6 model: i.e. rAG = rTC, rAC = rTG, rAT = rTA, rCG = rGC, rCA = rGT, rCT = rGA. The lnL of observing the data, D, given the tree, T, under the NREV6 model was calculated and stored for later comparisons. The model was then changed to the NREV12 model for which relative rates were defined such that it satisfied complete non-reversibility.

For each dataset we then used the lnL scores and numbers of free parameters in the three models for likelihood ratio tests (LRTs; (Anisimova, Bielawski, & Yang, 2001)) to determine whether (1) NREV12 fitted the data significantly better than GTR and (2) whether NREV12 fitted the data significantly better than NREV6. Specifically, for the NREV12 vs GTR comparison we calculated the LRTstatisticas2 (lnL_NREV12_ − lnL_GTR_) with the p value being calculated as 1 − chi (LRT, df_NREV12_ − df_GTR_). For the NREV12vsGTR comparison we calculated the LRTstatisticas2(lnL_NREV12_ − lnL_NREV6_) with the p value being calculated as 1 − chi (LRT, df_NREV12_ − df_NREV6_).

Further, the lnL scores and numbers of free parameters for each model were used to calculate AIC scores for each of the models(Posada & Buckley, 2004) which enabled us to identify which of the three models fit the data best. The model with the lowest AIC score was selected as the best fitting model with the AIC scores for the different models being calculated as follows:

(4) $$\text{AI}{\text{C}}_{\text{GTR}}=2\left(\text{d}{\text{f}}_{\text{GTR}}\right)-2\left(\ln{\text{L}}_{\text{GTR}}\right)\;\text{AI}{\text{C}}_{\text{NREV6}}=2\left(\text{d}{\text{f}}_{\text{NREV}}{}_{6}\right)-2\left(\ln{\text{L}}_{\text{NREV6}}\right),\;\text{AI}{\text{C}}_{\text{NREV12}}=2\left(\text{d}{\text{f}}_{\text{NREV12}}\right)-2\left(\ln{\text{L}}_{\text{NREV12}}\right).$$

Quantification of non-reversibility.

We further defined the degree of non-reversibility (DNR) as the absolute difference between the relative rate differences of two nucleotide pairs; i.e. for two nucleotides, *x* and *y*, there exists a relative rate of *x* to *y* substitutions that we will refer to as *m*, and a relative rate *y* to *x* substitutions that we will refer to as *n*. Under the NREV12 model, the degree of non-reversibility (DNR) between *x* and *y* is defined simply as the absolute difference between m and *n*:(|*m*-*n*|) and will hereby be referred to as *ij*_*DNR*_ were *i* and *j* are two nucleotides. We use DNR as a mathematical representation of the degree to which the rates of all pairs of reverse substitutions differ from one another. For each of 140 individual viral sequence datasets we calculated the average DNR of the six *ij*_*DNR*_ estimates inferred using the NREV12 model.

#### Simulations for testing the impact of non-reversible evolution on phylogenetic inference

We tested the accuracy of phylogenetic tree inference under reversible and non-reversible models using simulated datasets with varying average pairwise nucleotide sequence identities (APIs) evolved under the NREV12 model with different degrees of non-reversibility (DNR). The goal of these tests was not to exhaustively evaluate model misspecification issues during phylogenetic tree inference, but rather to check, in instances where viral taxa are known to be evolving in a detectably non-reversible manner (i.e. where NREV12 or NREV6 fits the data better than GTR), whether not accounting for this might decrease the accuracy of phylogenetic inference. Using IQTREE, a phylogenetic inference program that has the option to apply an NREV12-like model (referred to in IQTEE as the UNREST model), a phylogenetic tree was inferred from an alignment of real sequences (Avian Leukosis virus) with an average sequence identity (API) of ~ 90%. The branch lengths on this tree were then scaled to create four other phylogenetic trees representing sequences with approximately 95%, 85%, 80% and 75% API. These five trees are hereafter referred to as “true” trees and each individual tree was used as the starting point of a different set of simulations.

To test whether failure to account for non-reversibility might decrease the accuracy of phylogenetic inference we simulated the evolution of 5,500 nucleotide sequence alignments evolved non-reversibly under varying DNR along the five true phylogenetic trees: 100 datasets per true tree per simulated degree of non-reversibility (DNR). Specifically, simulations were done using HyPhy (Pond, Frost, & Muse, 2005) with relative rates ranging from a completely reversible matrix i.e., CA = AC = 0.166, GA = AG = 1, AT = TA = 0.14, GC = CG = 0.131, TG = GT = 0.188 and CT = TC = 1.101 – representing DNR = 0 – through matrices with DNR = 2, 4, 6, 8, 10, 12, 14, 16, 18 and 20 (Table 1). These baseline simulated substitution rates are reflective of those seen in empirical viral nucleotide sequence datasets.

At each DNR, the relative rates used conformed to standard measures of non-reversibility under the Kolmogorov conditions according to which non-reversibly evolving sequence datasets should yield three irreversibility indices (IRI1, IRI,2 and IRI3) that are all non-zero (Squartini & Arndt, 2008). Accordingly, whereas a for simulated datasets where DNR was 0, the IRI1, IRI2 and IRI3 indices were all approximately zero indicating that the sequences in these datasets had (as expected), evolved in a time-reversible manner, for datasets where DNR was greater than 0, the IRI1, IRI12 and IRI3 indices were all different from zero indicating that the sequences in these datasets had indeed evolved in a time non-reversible manner. Further, it should be noted that all simulations under NREV12 were performed under the stationarity criterion: $\pi {\text{e}}^{\text{Qt}}=\text{π}$ (where Q is the rate matrix and π is the nucleotide frequency distribution and t ≥ 0).

### Quantifying the accuracy of phylogenetic inferences.

We used the weighted Robinson-Foulds metric (wRF; implemented in the R phangorn package (Schliep, 2011)) to quantify differences between the true trees used to simulate datasets and the trees inferred from these datasets using the GTR or NREV12 models. wRF considers differences between both the topology and branch lengths of actual and inferred trees (Kuhner, 2015), (Robinson & Foulds, 1981).

## Results And Discussion

### Non-reversible nucleotide substitution models generally provide a better fit than reversible models to virus sequence datasets

We tested for evidence of non-reversibility of the nucleotide substitution process in 141 virus sequence datasets (33 ssDNA virus datasets, 30 dsDNA virus datasets, 31 dsRNA virus datasets, and 47 ssRNA virus datasets, all consisting of either full genome sequences (for unsegmented viruses), or complete genome component sequences (for viruses with segmented genomes). Specifically, for each dataset, we compared the goodness-of-fit of the GTR + G, NREV6 + G, and NREV12 + G models (where G represents gamma-distributed rates).

**Figure 2:**
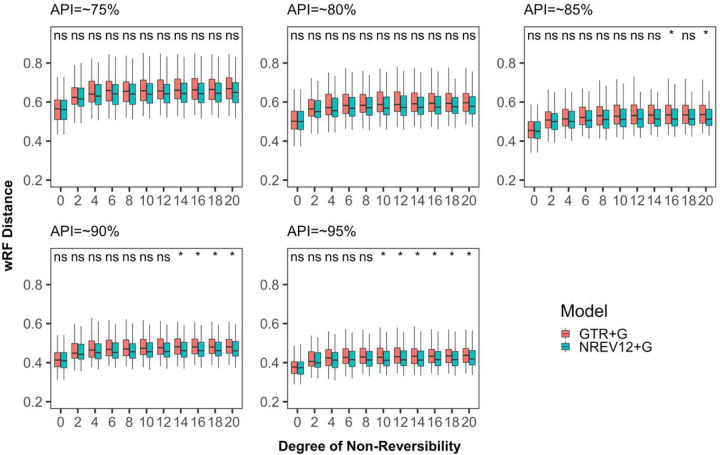
WeightedRobinson-Foulds distances between inferred and true phylogenetic trees for datasets simulated with different degrees of nucleotide substitution non-reversibility and different average pairwise sequence identities (APIs) (~75%%, ~80%, ~85%, ~90% and ~95%). ”ns” above a pair of box and whisker plots indicates a paired t-test adjusted p-value of greater than or equal to 0.05 and “*” indicate a paired t test adjusted p-value of <0.05

Given that dsDNA viruses such as adenoviruses, papillomaviruses and herpesviruses have both their DNA strands in existence for similar amounts of time before DNA-dependant-DNA polymerase enzymes copy both their + and – DNA strands during replication (Hanson, 2009), we had anticipated that the best fitting substitution model for sequence datasets of these viruses would be NREV6. Using weighted AIC scores to reveal trends of model support (Fig. 2), it is surprising that NREV12 was overall the best-supported model (illustrated by the redder hues around the top corner of the dsDNA plot in Fig. 2. Out of the 30 dsDNA datasets considered, we found that NREV6 provided the best fit to five datasets (HPV18, HPV45, HPV16, BPV, and SV40) and GTR provided the best fit to five (Alphapapilloma virus 6, JC polyomavirus, DPV, RTBV, and DBAV).NREV12 was the best fitting model for the remaining 20 datasets(Table 1). Further, LRTs revealed strong overall support for NREV12, with this model providing a significantly better fit (p < 0.05) than NREV6 for 25/30 of the dsDNA datasets and a significantly better fit than GTR for 24/30 of the datasets.

As NREV6 was not the best fitting model for most of the dsDNA virus datasets we infer that, in most dsDNA virus species, strand-specific substitution biases are not irrelevant. Further, the datasets where NREV6 was not the best fit are from species in families containing other species where NREV6 was the best fit, indicating that such strand-specific substitution biases are unlikely to be a consequence of some broadly conserved feature of viral life cycles in these families (such as, for example, ssDNA replicative intermediates). It is instead plausible that these differences may relate to:

- differences in replication fidelity and/or proofreading efficiency on the leading and trailing DNA strands in some dsDNA virus species (Grigoriev, 1999): These differences are common in eukaryotes (Youri, Newlon, & KunkelThomas, 2002) (Furusawa, 2012) and prokaryotes (Fijalkowska, Jonczyk, Tkaczyk, Bialoskorska, & Schaaper, 1998) and, considering that the replication processes of dsDNA viruses analyzed here mirror those of their eukaryote hosts, it is perhaps unsurprising that most of these viruses also display some evidence of strand-specific substitution biases.
- extra exposure to DNA damage of displaced template strands during unidirectional rolling circle replication in some papillomavirus species such as HPV16 could be a contributor to strand-specific nucleotide substitution biases (Kusumoto-Matsuo, Kanda, & Kukimoto, 2011).
- extra time spent by non-coding strands in single-stranded dissociated states during RNA transcription in some papillomavirus and polyomavirus species (Fernandes & de Medeiros Fernandes, 2012): during transcription processes, the dissociated non-coding strand is transiently more exposed to damage than the coding strand (Wei, et al., 2010) which might also contribute to strand-specific substitution biases.

Similarly, and equally surprising, we found that NREV12 was overall the best supported model for dsRNA viruses (illustrated by the redder hues around the top corner of the dsRNA plot in Fig. 2).

NREV6 fit only two of the 31 dsRNA datasets better than both NREV12 and GTR (Human rotavirus A set H and Fiji virus). NREV12 was found to be the best fitting model for 21/31 datasets and GTR was the best fitting of 8/31 (Table 2). In all three Birnaviridae family datasets (which contains virus species with two genome segments) and in 17/22 of Reoviridae family datasets (which contain virus species with 10–12 genome segments) the NREV12 model provided the best fit. Based on the LRTs, strong overall support for NREV12 was found, with this model providing a significantly better fit (p < 0.05) in 27/31 dsRNA virus datasets relative to NREV6, and 23/31 datasets relative to GTR.

We anticipated that NREV12 might fit many of these dsRNA datasets better than NREV6 simply because, during their infection cycles, the coding + strand of dsRNA viruses (the one from which protein translation occurs) tends to exist for longer periods within an infected cell than the non-coding –strand. Specifically, there are two main steps during double-stranded RNA virus replication (Wickner, 1993). Firstly, synthesis of the viral + strands from a dsRNA template occurs in the cytoplasm within viral particles. These + strands exist within the cell for prolonged periods in the absence of complementary -strands and are used as templates for translation of viral proteins. In the second step the + strands remaining after translation act as templates for -strand synthesis resulting in the formation of new dsRNA molecules. The + strands of dsRNA viruses are therefore likely more impacted by mutational processes, which in turn could explain the pervasive strand specific substitution biases seen in this group of viruses.

For the ssRNA and ssDNA viruses where one genome strand exists during the virus life cycle for far longer periods of time than the other such that complementary substitutions would not be expected to occur at similar rates, we anticipated that NREV12 should provide a better fit than both NREV6 and GTR. Indeed, for ssRNA viruses, NREV12 was a better fit than NREV6 and GTR for 40/47 of the ssRNA datasets and 24/33 of the ssDNA virus datasets (Fig. 2). Of the nine ssDNA virus datasets where NREV12 was not the best fitting model, GTR fit 7/9 better and NREV6 fit 2/9 better (Table 3). Of the seven ssRNA datasets (Table 4) where NREV12 was not the best fitting model, GTR fit 6/7 better and NREV6 fit 1/7 better.

Based on the LRTs, strong overall support for NREV12 was found with this model providing a significantly better fit (p < 0.05) than NREV6 for 45/47 of the ssRNA virus datasets (Table 4) and 31/33 of the ssDNA virus datasets (Table 3). Similarly, based on LRTs, NREV12 provided a significantly better fit than GTR for 27/33 of the ssDNA virus datasets (Table 3) and 40/47 of the ssRNA virus datasets (Table 4).

We found that DNR estimates alone did not cleanly differentiate between datasets for which NREV12 was or was not best supported (Tables 1, 2, 3 and 4). For the 107 nucleotide sequence datasets with a model preference of NREV12, ten had estimated DNRs that were greater than 0.5, 13 had DNRs between 0.25 and 0.5 and 84 had DNRs between 0.0225 and 0.25. For the ten nucleotide sequence datasets with a model preference of NREV6, one had an estimated DNR greater than 0.5, four had estimated DNRs between 0.25 and 0.5 and five had estimated DNRs between 0.064 and 0.25 (Fig. 2). For the 24 nucleotide sequence datasets with a model preference of GTR, none had estimated DNRs greater than 0.5, one had an estimated DNR between 0.25 and 0.5 and the remainder had estimated DNRs between 0.0335 and 0.25.

The dsDNA virus dataset with the highest DNR was Human mastadenovirus D (DNR = 0.646), the dsRNA virus dataset with the highest estimated DNR was Porcine_rotavirus_B (0.351), the ssRNA virus dataset with the highest DNR was SARS-CoV-2 (DNR = 1.536) and the ssDNA virus dataset with the highest DNR was Torque teno sus virus (DNR = 1.56).

Therefore, while NREV12 appears to be generally more appropriate than either NREV6 or GTR for describing mutational processes in ssRNA, ssDNA, dsDNA, and dsRNA viruses, this might only be particularly relevant from a practical perspective when datasets of these viruses yield DNR estimates that are greater than 0.25. For such datasets NREV12 (and possibly NREV 6 in some instances) might be especially useful for both determining the direction of evolution across phylogenetic trees (i.e., it could potentially be used to root these trees) and for quantifying genomic strand-specific nucleotide substitution biases (Harkins et al., 2009).

### Assessing the impacts of model misspecification on phylogenetic tree inference

To determine whether it might sometimes be worthwhile using NREV12 rather than GTR for phylogenetic inference when NREV12 is the best fitting nucleotide substitution model, we used simulated datasets to compare the accuracy of phylogenetic trees inferred using these models. Specifically, we simulated datasets with DNRs varying from 0 to 20 along known “true” phylogenetic trees (supplementary Figure S 1) with branches scaled to reflect branch tip sequences with APIs of ~ 75%, ~ 80%, ~ 85%, ~ 90% and ~ 95%. For each of five API levels, we therefore simulated 5500 datasets (comprising 100 datasets for each DNR = 0, 2, 4, 6, 8, 10, 12, 14, 16, 18 and 20).

Phylogenetic trees were inferred from these 5500 simulated datasets and compared to the phylogenetic trees used to simulate the datasets (i.e., the true trees) using wRF distances to assess the impact of varying DNR on the accuracy of phylogenetic inference. We further tested whether the accuracy of phylogenetic inference could be improved for sequences that had evolved under DNR > 0 by using NREV12 instead of GTR. Specifically, for every simulated dataset a phylogenetic tree was inferred using GTR and another using NREV12 and the wRF distances of each of these trees to the true tree was determined. For each of the analysed DNRs a paired t-test (correlated t-test) was then used to compare the wRF scores of trees inferred using GTR and NREV12. We were particularly interested in determining whether trees inferred using a mis-specified model (i.e. GTR in this case) would be less accurate than trees inferred with a correctly specified model (i.e. NREV12).

We found that, irrespective of dataset diversity and the nucleotide substitution model used, phylogenetic inference tended to become more inaccurate (i.e. wRF scores increased) as DNR increased (Fig. 2). This tendency was, however, more pronounced when using a (miss-specified) GTR model than when using a (correctly specified) NREV12 model with, for any given dataset having DNR > 0, the use of NREV12 tending to yield more accurate phylogenetic trees than when GTR was used. There were, however, only statistically significant improvements (p < 0.05 paired t-test) in the accuracy of phylogenetic trees inferred using NREV12 relative to those inferred using GTR in lower diversity datasets (i.e. those with APIs of 85%, 90% and 95%); and then only for DNR > 8. It is noteworthy that the highest estimated DNR in any of the empirical datasets that we analysed was xxx: more than four-fold lower than the point where statistically significant differences in phylogenetic-inference accuracies became apparent in the simulated datasets.

## Conclusion

The non-reversible nucleotide substitution model, NREV12, generally provides a substantially better fit to virus nucleotide sequence datasets than does the widely used reversible substitution model, GTR. NREV12 also generally provides a better fit to virus nucleotide sequence datasets than does NREV6; a non-reversible model that would be expected to best describe the evolution of double stranded genome sequences that display no strand-specific nucleotide substitution biases. This suggests that, contrary to our expectations, substantial strand-specific nucleotide substitution biases (i.e. estimated DNRs > 0.25) are common during viral evolution irrespective of genome type. Such biases should be expected for any viruses where one genome strand either is in existence for substantially longer periods of time than the other, or is more exposed to mutagenic processes than the other during transmission, replication, or gene expression.

We had anticipated that, given evidence of sequences evolving both non-reversibly and with strand-specific substitution biases, inferring trees using a model such as NREV12 that appropriately accounts for this might: (1) minimize the impact of increasing DNR on the accuracy of phylogenetic inference (i.e. wRF scores presented in blue in Fig. 2 might have been expected to not increase with increasing DNR); and (2) yield significantly more accurate phylogenetic inferences than when using GTR for all datasets where NREV12 was the most appropriate model and DNRs were greater than zero. However, increasing DNR clearly decreased the accuracy of phylogenetic inference even when using NREV12, and, for datasets where DNRs were greater than zero, using GTR did not consistently yield significantly less accurate phylogenetic inferences than those attained using NREV12. From a practical perspective, choosing a non-reversible nucleotide substitution model to construct phylogenetic trees from virus genome sequences that display strand specific nucleotide substitution biases is not guaranteed to yield more accurate phylogenetic trees. Nevertheless, in instances where strand specific substitution biases are higher than ~ 0.5 (such as are found in our SARS-CoV-2,, Torque teno sus virus and Banana bunchy top virus datasets) it may be prudent to select a model such as NREV12 (such as is implemented in programs like IQTREE) over GTR as the better of two suboptimal choices.

The lack of available data regarding the proportions of viral life cycles during which genomes exist in single and double stranded states makes it difficult to rationally predict the situations where the use of models such as GTR, NREV6 and NREV12 might be most justified: particularly in light of the poor over-all performance of NREV6 and GTR relative to NREV12 with respect to describing mutational processes in viral genome sequence datasets. We therefore recommend case-by-case assessments of NREV12 vs NREV6 vs GTR model fit when deciding whether it is appropriate to consider the application of non-reversible models for phylogenetic inference and/or phylogenetic model-based analyses such as those intended to test for evidence of natural section or the existence of molecular clocks.

## Supplementary Material

Supplement 1

## Figures and Tables

**Figure 1:**
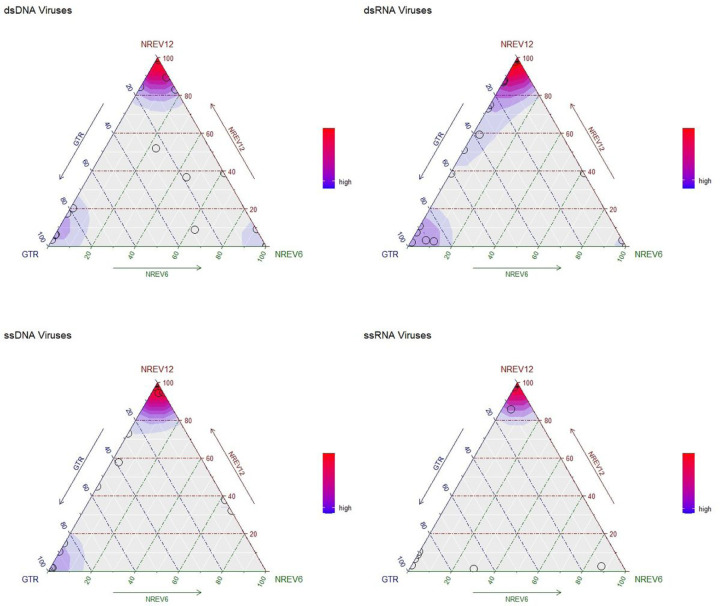
Ternary plots illustrating the relative fit of the NREV12, NREV6, and GTR nucleotide substitution models based on weighted AIC scores for 30 dsDNA, 31 dsRNA, 33 ssDNA, and 47 ssRNA virus nucleotide sequence datasets. These plots were produced using the Akaike weights function with an overlaid density function (implemented in the qpcR package of RStudio (Ritz & Spiess, 2008) to indicate point densities. Each model is represented by a corner of the triangles, and each circle represents the relative fit of each of the three models to a single nucleotide sequence dataset. The sides of the triangle represent model support axes ranging from 0–100%, with the position of a circle in relation to each of the sides of the triangle indicating the probability of models best describing the nucleotide sequence dataset that is represented by that point. Whereas strong red colours represent a very high density of nucleotide sequence datasets that favour a particular model, bluer colours indicate a lower, but still substantial, density of datasets that favour a model.

**Table 1:** Relative rate change for C to A, G to A, A to T, G to C, T to G and C to T mutations under the 11 degrees of non-reversibility alongside the maintained rates for A to C, A to G, T to A, C to G, G to T, and T to C

Degree of Non-Reversibility (DNR)	Relative rates of different nucleotide substitution types (from-to)											
	C-A	A-C	G-A	A-G	A-T	T-A	G-C	C-G	T-G	G-T	C-T	T-C
0	0.166	0.166	1	1	0.14	0.14	0.131	0.131	0.118	0.118	1.101	1.101
2	2.166	0.166	3	1	2.14	0.14	2.131	0.131	2.118	0.118	3.101	1.101
4	4.166	0.166	5	1	4.14	0.14	4.131	0.131	4.118	0.118	5.101	1.101
6	6.166	0.166	7	1	6.14	0.14	6.131	0.131	6.118	0.118	7.101	1.101
8	8.166	0.166	9	1	8.14	0.14	8.131	0.131	8.118	0.118	9.101	1.101
10	10.166	0.166	11	1	10.14	0.14	10.131	0.131	10.118	0.118	11.101	1.101
12	12.166	0.166	13	1	12.14	0.14	12.131	0.131	12.118	0.118	13.101	1.101
14	14.166	0.166	15	1	14.14	0.14	14.131	0.131	14.118	0.118	15.101	1.101
16	16.166	0.166	17	1	16.14	0.14	16.131	0.131	16.118	0.118	17.101	1.101
18	18.166	0.166	19	1	18.14	0.14	18.131	0.131	18.118	0.118	19.101	1.101
20	20.166	0.166	21	1	20.14	0.14	20.131	0.131	20.118	0.118	21.101	1.101

**Table 1:** AIC Scores and LRT results for double-stranded DNA virus datasets. The lowest AIC scores indicating the best-fitting models are in bold

Virus Family	Dataset	AIC Score GTR	AIC Score NREV-6	AIC Score NREV-12	P-Value GTR vs NREV-12	P-Value NREV-6 vs NREV-12	DNR
Papillomaviridae	APPV 6	**35099.5**	35108.0	35102.2	> 0.05	0.007	0.089
	HPV18_2	25202.9	**25174.6**	25179.2	< 0.001	> 0.05	0.323
	HPV45_2	23600.6	**23599.0**	23602.9	> 0.05	> 0.05	0.285
	HPV16_2	29734.0	**29664.5**	29665.4	< 0.001	> 0.05	0.371
	HPV31	24681.4	24677.3	**24672.8**	0.002	0.01	0.165
	HPV6_1	31199.1	31150.0	**31141.2**	< 0.001	< 0.001	0.451
	LPV	67165.7	67188.1	**67145.5**	< 0.001	< 0.001	0.42
	DPV	**69829.7**	69889.2	69835.1	> 0.05	< 0.001	0.056
	XPV	95455.6	95617.1	**95452.2**	< 0.001	< 0.001	0.072
	BATV	134821	134511	**133322**	< 0.001	< 0.001	0.402
Polyomaviridae	JC_2	**51806.7**	51819.6	51812.0	> 0.05	0.003	0.089
	BK_2	21472.6	21472.7	**21471.1**	0.03	0.03	0.244
	SV40	16859.8	**16858.0**	16858.4	0.037	> 0.05	0.567
	BPV	148614.9	**148573.8**	148585.2	< 0.001	> 0.05	0.064
Caulimoviridae	CMV	124083.9	124221.0	**123888.6**	< 0.001	< 0.001	0.351
	CSSV	145327.0	146575	**145202**	< 0.001	< 0.001	0.158
	SVBV	138575	138488.1	**138464.7**	< 0.001	< 0.001	0.174
	DBAV	**46495.5**	46514.1	46502.0	> 0.05	< 0.001	0.0335
	RTBV	**54987.9**	55350.1	54991	> 0.05	< 0.001	0.082
	BDV	376325.2	376647.6	**376029.9**	< 0.001	< 0.001	0.140
Siphoviridae	CLV	237362.3	237351.8	**237348.6**	< 0.001	< 0.01	0.070
Tectiviridae	TTIV	913864.9	913915.4	**913773.1**	< 0.001	< 0.001	0.279
Adenoviridae	FAV_C	3074086.7	3074207.5	**3073739.1**	< 0.001	< 0.001	0.169
	FAV_E	103482.3	103222.7	**102636.7**	< 0.001	< 0.001	0.357
	FAV_D	2326925.6	2325719.4	**2324784.5**	< 0.001	< 0.001	0.551
	FAV_A	705328.5	705436..5	**705197.8**	< 0.001	< 0.001	0.645
	HMAV_B	103796.7	103937.44	**103753.8**	< 0.001	< 0.001	10.890
	HMAV_D	1748635.2	1749769	**1748119.1**	< 0.001	< 0.001	0.646
	HMAV_C	2851144.5	2851357.1	**2851133**	0.006	< 0.001	0.0225
	HMAV_E	1915044.8	1915065.3	**1914998**	< 0.001	< 0.001	0.049

**Table 2:** AIC Scores and LRT results for double-stranded RNA datasets. The lowest AIC scores indicating the best fitting models are in bold.

Virus Family	Dataset	AIC score GTR	AIC score NREV-6	AIC score NREV-12	GTR vs NREV-12	NREV-6 vs NREV-12	DNR
Birnaviridae	AQBV	31754.9	31853.3	**31721.9**	< 0.001	< 0.001	0.219
	GBV_A	47176.9	47347.2	**47154.8**	< 0.001	< 0.001	0.142
	IPNV	79186.2	79221.9	**79182.4**	0.0145	< 0.001	0.162
	GBV_B	39313.7	39062.8	**38938.7**	< 0.001	< 0.001	0.201
Reoviridae	BTV_A	34803.5	34895.1	**34801.3**	0.03	< 0.001	0.042
	BTV_B	48849.9	48893.	**48837.1**	< 0.001	< 0.001	0.043
	BTV_C	28350.9	28386.5	**28350.8**	> 0.05	< 0.001	0.061
	BTV_D	24969.1	24947.3	**24894.0**	< 0.001	< 0.001	0.191
	BTV_F	20622.7	20708.5	**20610.2**	< 0.001	< 0.001	0.067
	BTV_G	63349.9	63485.0	**63345.9**	0.00426	< 0.001	0.040
	BTV_H	20596.7	20685.5	**20586.1**	< 0.001	< 0.001	0.118
	BTV_I	17592.7	17622.5	**17588.8**	0.01	< 0.001	0.095
	BRVA_C	41206.7	41187.4	**41137.1**	< 0.001	< 0.001	0.128
	HRVA_A	**17030.5**	17043.2	17035.5	> 0.05	0.003	0.036
	HRVA_B	**8275.1**	8280.3	8281.7	> 0.05	> 0.05	0.087
	HRVA_C	12815.1	12842.6	**12807.6**	0.003	< 0.001	0.132
	HRVA_D2	**8036.8**	8041.0	8043.7	> 0.05	> 0.05	0.057
	HRVA_E	**7045.9**	7056.1	7053.3	> 0.05	0.02	0.102
	HRVA_F	**7046.0**	7056.7	7053.4	> 0.05	0.02	0.0710
	HRVA_G	18424.2	18434.0	18425.1	> 0.05	< 0.001	0.123
	HRVA_H	20431.4	**20413.87**	20420.5.6	0.002	> 0.05	0.163
	PRVA_A	28540.7	28441.9	**28398.7**	< 0.001	< 0.001	0.204
	PRVA_B	14757.7	14775.5	**14732.6**	< 0.001	< 0.001	0.351
	HRVC_A	6713.2	6718.2	**6712.3**	0.045	0.007	0.124
	PTOV	202011.3	202106.5	**201878.5**	< 0.001	< 0.001	0.039
	FJV_B	9274.1	**9250.0**	9250.9	< 0.001	> 0.05	0.194
Endornaviridae	EDV	1771992.8	1772689.1	1771950.6	< 0.001	< 0.001	0.121
	BPAV	**70386.5**	70540.2	70390.7	> 0.05	0.00	0.047
Totiviridae	TTV	617302.6	617462.6	**617172.9**	< 0.001	< 0.001	0.052
	GDV	**80435.8**	80396.5	80387.7	< 0.001	0.002	0.109
Hypoviridae	HPV	66859.8	66899.8	**66857.8**	0.03	< 0.001	0.057

**Table 3:** AIC Scores and LRT results for single-stranded DNA datasets. The lowest AIC scores indicating the best fitting models are in bold.

Virus Family	Dataset	AIC Score GTR	AIC Score NREV-6	AIC Score NREV-12	P-Value GTR vs NREV-12	P-Value NREV-6 vs NREV-12	DNR
Nanoviridae	BBTV M	15044.3	15207.9	**14984.4**	< 0.001	< 0.001	0.662
	BBTV N	10605.6	10686.2	**10595.2**	< 0.001	< 0.001	0.533
	BBTV R	18484.5	18544	**18480.8**	> 0.05	< 0.001	0.609
	BBTV S	12718.9	12757.2	**12707.3**	< 0.001	< 0.001	0.728
	CCDV	**38622.7**	38632.0	38630.5	> 0.05	0.03	0.050
	MDV	36232.8	**36063**	36064	< 0.001	> 0.05	0.142
	PYDV	56138.4	56076.6	**56056.4**	< 0.001	< 0.001	0.187
	FBNS	100153.6	100135.6	**100120.5**	< 0.001	< 0.001	0.098
Geminiviridae	Begomo 5	**28192.1**	28311.9	28192.5	> 0.05	< 0.001	0.1995
	Begomo 6	16743.0	**16722.6**	16724.1	< 0.001	> 0.05	0.214
	Begomo 9	8517.6	8540.8	**8515.6**	0.03	< 0.001	0.312
	Dicot 1	44730.7	44594.3	**44583.3**	< 0.001	< 0.001	0.200
	Dicot 2	**39909.9**	39919.8	39917.9	> 0.05	< 0.001	0.100
	MSV	**252645.3**	254347.5	254347.5	< 0.001	< 0.001	0.144
	PanSV	94601.2	94600.3	**94593.7**	< 0.001	< 0.001	0.182
	WDV	35301.7	35313.2	**35253.8**	< 0.001	< 0.001	0.1033
Circoviridae	BFDV	17256.7	17262.7	**17246.7**	< 0.001	< 0.001	0.224
	DG_CV	**12754.8**	12779.5	12758.3	> 0.05	< 0.001	0.116
	PiCV	**19180.5**	19192.5	**19191.0**	> 0.05	0.04	0.117
	CCCC	84435.7	84377.4	**84315.3**	< 0.001	< 0.001	0.132
	BTC	262910.4	262060.1	**261985.4**	< 0.001	< 0.001	0.178
	POCV2	24940.9	24953.8	**24915.8**	< 0.001	< 0.001	0.162
	CCV	90307.9	90301.5	**90285.9**	< 0.001	< 0.001	0.114
Anelloviridae	TTV_1	825811	826800	**825292**	< 0.001	< 0.001	0.513
	TTSV	332287.9	332397.4	**332258.2**	< 0.001	< 0.001	1.560
Parvoviridae	MVM	26756.3	26743.9	**26686.9**	< 0.001	< 0.001	0.148
	HPV	67051.2	67080.1	**67001.8**	< 0.001	< 0.001	0.235
	CPV	85731	85695	**85689.3**	< 0.001	0.007	0.062
	PPV	163006.8	163090.7	**162995.9**	< 0.001	< 0.001	0.143
	CAV_P	37073.3	37115.5	**37065.7**	< 0.001	< 0.001	0.162
Microviridae	BMV	31175.3	31164.8	**31147.3**	< 0.001	< 0.001	0.188
Pleolipoviridae	APV	85700.2	85617.4	**85402.8**	< 0.001	< 0.001	0.204
	BPV	204797.5	204802.3	**204796.7**	0.04	0.007	0.064

**Table 4:** AIC Scores and LRT results for single-stranded RNA datasets. The lowest AIC scores indicating the best fitting models are in bold.

Virus Family	Dataset	AIC Score GTR	AIC Score NREV-6	AIC Score NREV-12	P-Value GTR vs NREV-12	P-Value NREV-6 vs NREV-12	DNR
Astroviridae	HAV	94580.7	94926.3	**94548.1**	< 0.001	< 0.001	0.096
	BAV	188307.1	188572.9	**188144.9**	< 0.001	< 0.001	0.108
	MMV	281072.2	281094.5	**281076.9**	> 0.05	< 0.001	0.072
	PAV	150626.88	150827.6	**150609.5**	< 0.001	< 0.001	0.069
	CKV	90902.3	91233.1	**90873.0**	< 0.001	< 0.001	0.083
	GA	64998.5	65223.9	**64975.9**	< 0.001	< 0.001	0.110
	CAV_A	85558.8	85617.4	**85547.3**	< 0.01	< 0.001	0.076
Bromoviridae	CMV RNA1	34197.5	34198.8	**34147.7**	< 0.001	< 0.001	0.124
	CMV RNA2	**31398.2**	31455.9	31388.7	< 0.001	< 0.001	0.091
	CMV RNA3	**24337.2**	24360.3	24343.9	> 0.05	< 0.001	0.073
	AMS	**24337.2**	24360.3	24343.9	> 0.05	< 0.001	0.073
	PSV	67707	67786.5	**67691**	< 0.001	< 0.001	0.048
Caliciviridae	LAV	73042.8	73102.4	**72984.6**	< 0.001	< 0.001	0.120
	NoV	207667.2	207777.5	**207660**	< 0.001	< 0.001	0.047
	VSV	235936.4	236051.4	**235913.3**	< 0.001	< 0.001	0.046
Closteroviridae	CTV	**30062.2**	29980.4	29960.1	< 0.001	< 0.001	0.272
Flaviviridae	DGV_T1	69771.9	70030.5	**69776.2**	> 0.05	< 0.001	0.063
	JEV	146920.8	148101.5	**146885.5**	< 0.001	< 0.001	0.091
Hepeviridae	HPVE1	200439.5	200863.8	**200179.8**	< 0.001	< 0.001	0.073
	HPVE2	155709.1	155983.8	**155518.6**	< 0.001	< 0.001	0.088
Picornaviridae	ENV_A	552287.9	553535.5	**551794.1**	< 0.001	< 0.001	0.061
	HRV_A	102218.7	102267.0	**101550.7**	< 0.001	< 0.001	0.285
	AIV	101073.1	101136.7	**101052.2**	< 0.001	< 0.001	0.093
	AHP	139635.7	140119.6	**139506.9**	< 0.001	< 0.001	0.170
	ECV	82078.9	82181.0	**82065.8**	< 0.001	< 0.001	0.066
	CDV	130551.3	130896.7	**130478.3**	< 0.001	< 0.001	0.086
	TCV	53027.3	53029	**53023**	0.0151	0.0422	0.033
	FMDV	455180.6	455582.6	**454806.1**	< 0.001	< 0.001	0.117
Fusariviridae	FRV	52413.1	52470.6	52418.4	> 0.05	< 0.001	0.076
Retroviridae	HIV1_setA	344014.4	344295.1	**343669.7**	< 0.001	< 0.001	0.237
	HIV1_M	80764.1	80829.5	**80668.1**	< 0.001	< 0.001	0.442
	HIV1_setC	180575.0	180702.3	**180494.4**	< 0.001	< 0.001	0.107
	HIV1_setD	298489.9	298695.3	**298260.6**	< 0.001	< 0.001	0.133
	HIV1_setE	289111.3	289292.1	**288941.9**	< 0.001	< 0.001	0.112
	HIV1_setF	214375.9	214692.2	**214289.4**	< 0.001	< 0.001	0.148
	EIV	126149	126365.4	**125300**	< 0.001	< 0.001	0.192
	BIV	**24505.2**	24506.9	24513.2	> 0.05	> 0.05	0.15
	FIV	164542.1	164487.9	**164260.4**	< 0.001	< 0.001	0.114
	CAV	351329.9	351871.5	**350721.9**	< 0.001	< 0.001	0.174
	SIV	110731.2	110816	**110663.3**	< 0.001	< 0.001	0.144
Filoviridae	Ebola_2	53147.3	**53143.0**	53149.9	> 0.05	> 0.50	0.264
Orthomyxoviridae	Flu A 2	82872.8	83010.2	**82849.7**	< 0.001	< 0.001	0.27
	Flu B 1	50090.4	50144.1	**50060.9**	< 0.001	< 0.001	0.311
Coronaviridae	SARS-COV1	214715.3	214968.5	**214644.39**	< 0.001	< 0.001	0.198
	SARS-COV2	15715.4.2	15715.6	**15696.7**	< 0.001	< 0.001	1.536
	SARB	573966.3	573815.1	**572517.0**	< 0.001	< 0.001	0.301
	MERS-COV	516683.2	516983.4	**516608.9**	< 0.001	< 0.001	0.169

## Abbreviations

API: Average pairwise nucleotide sequence identity
NREV6: 6 Rate Non-Reversible Model
NREV12: 12 Rate Non-Reversible Model
GTR: General time Reversible Model
dsDNA: Double-stranded deoxyribonucleic acid
dsRNA: Double-strandedribonucleic acid
ssDNA: Single-stranded deoxyribonucleic acid
ssRNA: Single-strandedribonucleic acid
DNR: Degrees of Non-Reversibility
wRF: Weighted Robinson-Foulds distance
LRT: likelihood ratio test
AIC: Akaike information criterion test
