# Supplementary material for: Viral genome sequence datasets display pervasive evidence of strand-specific substitution biases that are best described using non-reversible nucleotide substitution models
Source: Res Sq. 2022 Dec 29:rs.3.rs-2407778. Preprint. [Version 1] doi: 10.21203/rs.3.rs-2407778/v1 (PMC9810213; doi:10.21203/rs.3.rs-2407778/v1)
Supplement: Supplementary file 1 [file NIHPPRS2407778v1-supplement-1.pdf]

Supplementary Files

This is a list of supplementary files associated with this preprint. Click to download.

- SupplementaryFigureAndTable.docx
